# Elderly patient with unresectable advanced‑stage hepatocellular carcinoma who received atezolizumab plus bevacizumab and achieved a complete response: A case report

**DOI:** 10.3892/mi.2024.147

**Published:** 2024-03-20

**Authors:** Shuhei Arima, Tatsuo Kanda, Mai Totsuka, Masayuki Honda, Shini Kanezawa, Reina Sasaki-Tanaka, Naoki Matsumoto, Ryota Masuzaki, Hiroaki Yamagami, Masahiro Ogawa, Hirofumi Kogure

**Affiliations:** Division of Gastroenterology and Hepatology, Department of Medicine, Nihon University School of Medicine, Tokyo 137-8610, Japan

**Keywords:** older patient, liver cancer, stage IV disease, systemic therapy, complete response

## Abstract

Hepatocellular carcinoma (HCC) is a common malignancy with a poor prognosis, particularly in patients with advanced-stage disease, elderly individuals and/or in those with poor liver function. Immune checkpoint inhibitor-containing therapies, such as atezolizumab, an anti-programmed death ligand-1 monoclonal antibody, plus bevacizumab, an anti-vascular endothelial growth factor monoclonal antibody, may be effective and safe therapeutic options for elderly patients with advanced-stage HCC. The present study reports the case of a male patient his 80s who consumed alcohol with unresectable advanced-stage HCC who received combination therapy comprising atezolizumab plus bevacizumab for 6 months. The patient achieved a complete response despite the discontinuation of treatment due to nephrotoxicity. It is critical for patients with HCC and a Child-Pugh A grade to continue therapy for HCC, even if they are older. The development of more effective therapies is required for patients with advanced-stage HCC with a worse liver function than those with a Child-Pugh A grade. The case described in the present study demonstrates the need for obtaining further evidence regarding the efficacy and safety of the combination therapy including atezolizumab plus bevacizumab for elderly patients with advanced-stage HCC.

## Introduction

The treatment algorithm of the Asian Pacific Association for the Study of the Liver (APASL) recommends systemic therapy or best supportive care (BSC) for patients classified as Child-Pugh A/B or C with hepatocellular carcinoma (HCC) and extrahepatic metastasis, respectively (1). The European Association for the Study of the Liver (EASL) recommends systemic therapy or BSC for patients with cirrhosis with advanced- or terminal-stage HCC (2). The American Association for the Study of Liver Diseases (AASLD) recommends the use of systemic therapy over no therapy for patients with Child-Pugh A liver cirrhosis or for well-selected patients with Child-Pugh B liver cirrhosis plus advanced-stage HCC with macrovascular invasion and/or metastatic disease (3).

Although the multi-kinase inhibitors, sorafenib and lenvatinib, are approved first-line systemic treatments for unresectable HCC, treatment with atezolizumab [an anti-programmed death ligand-1 (PD-L1) monoclonal antibody] plus bevacizumab (an anti-vascular endothelial growth factor monoclonal antibody) (4) or tremelimumab (an anti-cytotoxic T-lymphocyte-associated antigen-4 monoclonal antibody) plus durvalumab (an anti-PD-L-1 monoclonal antibody) (5) is currently used as a first-line systemic therapy for patients with HCC classified as Child-Pugh A in Japan. In general, patients with unresectable HCC have a poor prognosis (6).

The long-term prognosis of elderly patients with HCC following hepatic resection is determined by the presence of liver cirrhosis and vascular invasion (7). Increased mortality following hepatectomy has been shown to be significantly associated with an older age (8). An older age and cardiac comorbidity have been shown to be significantly associated with radiofrequency ablation-related mortality (8). Another study demonstrated that the efficacy and safety of sorafenib did not differ significantly between younger and elderly patients with HCC (9). At present, researchers have not yet determined whether immune checkpoint inhibitor (ICI)-containing therapies, such as atezolizumab plus bevacizumab, are effective and safe for use in elderly patients with advanced-stage HCC (10,11). Treatment with ICIs can increase the risk of multi-organ autoimmune inflammatory responses, which are the most common type of complication associated with this type of treatment (12).

The present study reports the case of an elderly patient with unresectable advanced-stage HCC who received atezolizumab plus bevacizumab and achieved a complete response (CR) based on the modified Response Evaluation Criteria in Solid Tumors (mRECIST) (13,14).

## Case report

A male patient in his 80s who consumed alcohol experienced body weight loss (-10 kg over a period of 6 months) in addition to appetite loss 2 months prior. He was regularly examined by a local doctor for hypertension or diabetes mellitus and was being treated with 10 mg empagliflozin, 5 mg linagliptin, 50 mg vildagliptin and 0.75 mg repaglinide daily, or 50 mg losartan potassium daily. The patient was subsequently introduced to Nihon University Itabashi Hospital (Tokyo, Japan) due to multiple liver tumors and suspected lung tumors in the right apex area.

Of note, 10 and 5 years prior, the patient had undergone cataract surgery and had spinal canal stenosis, respectively. In addition, 2 years prior to his admittance to the local hospital, he had received a transfusion for colonic diverticular bleeding. The patient did not have any tattoos or any history of drug abuse, and he consumed one cup of alcohol daily.

At the time of the first visit, the body length and body weight of the patient were 172 cm and 65 kg, respectively. His blood pressure, pulse rate, O_2_ saturation and body temperature were 169/67 mmHg, 62/min, 97% and 36.4˚C, respectively. He was conscious, his conjunctiva were not icteric or anemic, and liver tumors were palpable in the right hypochondriac region. No edema was observed on the feet. The Eastern Cooperative Oncology Group (ECOG) performance status score was 1 (PS-1).

The laboratory data from the first visit are presented in Table I and indicated mild abnormalities in liver function. The Child-Pugh score and grade were 5 and A, respectively. The albumin-bilirubin (ALBI) score was Grade 2a. Tests for both hepatitis B surface antigen and anti-hepatitis C virus antibodies yielded negative results. Although a history of hepatitis B virus (HBV) infection was suggested, serum HBV DNA was not detected. The α-fetoprotein (AFP) and lectin-reactive protein (AFP-L3) levels were elevated. Diabetes mellitus was well controlled.

**Figure 1:**
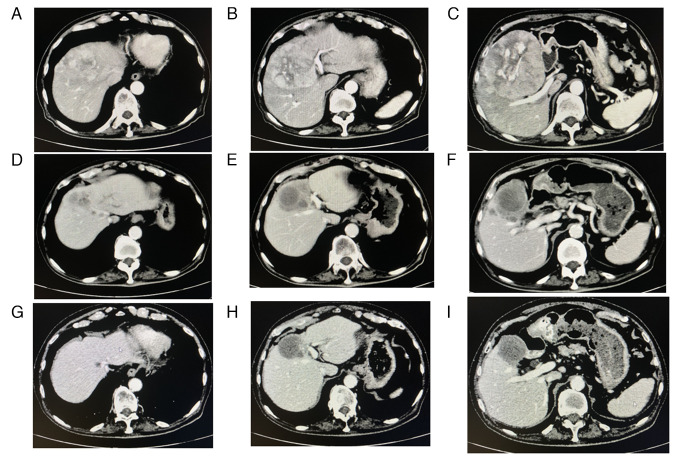
Changes in abdominal CT scan images obtained during combination therapy with atezolizumab plus bevacizumab. (A-C) Prior to therapy, a CT scan demonstrated multiple hepatocellular carcinomas in the liver and swelling of the lymph nodes in the paraaortic lesion and other sites. (D-F) After 5 months of combination therapy, a CT scan demonstrated the disappearance of any intratumoral arterial enhancement in any of the target lesions. (G-I) At 4 months after the combination treatment was suspended, a CT scan did not demonstrate any recurrences. CT, computed tomography.

A pre-treatment abdominal computed tomography (CT) scan demonstrated that 110 mm and other HCC nodules were present in segment 4 (S4), S5 and S8 of the liver, respectively. The P4 portal vein was disrupted, and swelling of the lymph nodes was observed in the paraaortic lesion and other sites (Fig. 1A-C).

The patient was diagnosed with HCC stage IVA (15) and alcohol-associated liver cirrhosis with diabetes mellitus and hypertension. After screening for autoimmune diseases and/or the presence of high-risk esophageal varices, 1,200 mg atezolizumab (Tecentriq; Chugai Pharmaceutical Co., Ltd.) was administered intravenously, followed by 900 mg bevacizumab (Avastin; Chugai Pharmaceutical Co., Ltd.) every 3-4 weeks.

When unacceptable grade 2 or 3 adverse events developed, the combination therapy was suspended. Based on the mRECIST criteria (13,14), tumor assessment was performed by a contrast-enhanced CT scan every 1-2 months.

**Figure 2:**
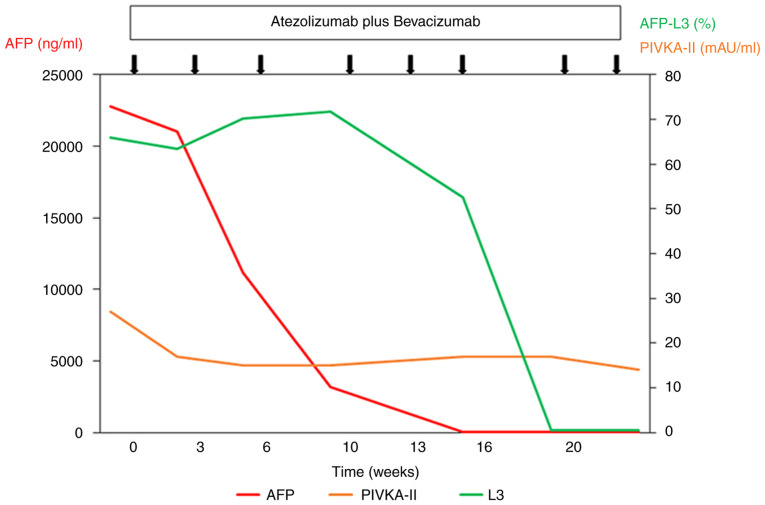
Clinical course and changes in tumor markers in hepatocellular carcinoma in the case described herein. At 5 months after the commencement of combination therapy comprising atezolizumab and bevacizumab, the tumor marker levels of the patient returned to within normal limits. AFP, α-fetoprotein; AFP-L3, lectin-reactive profile of AFP; PIVKA-II, protein induced by vitamin K absence or antagonist II. The black arrows indicate the time points of single combination therapy with atezolizumab and bevacizumab.

At 5 months following the commencement of the combination therapy, when six cycles of combination therapy were completed, the tumor marker levels of the patient returned to within normal limits: AFP, 1.8 ng/ml; AFP-L3, <0.5%; and protein induced by vitamin K absence or antagonist II (PIVKA-II), 17 mAU/ml (Fig. 2). A contrast-enhanced CT scan demonstrated the disappearance of any intratumoral arterial enhancement in any of the target lesions, indicating that the treatment response was complete (Fig. 1D-F). After 6 months of combination treatment with atezolizumab plus bevacizumab, the combination treatment was terminated due to the accidental occurrence of interstitial nephritis. The patient received steroid therapy and recovered.

When eight cycles of combination therapy were completed after 6 months of commencement, the patient terminated the combination therapy of atezolizumab plus bevacizumab due to his leg edema and renal dysfunction. His serum creatinine, albumin levels and estimated glomerular filtration rates (eGFR) were 1.86 mg/dl, 2.9 g/dl and 28 ml/min/1.73 m^2^, respectively. Urinary protein, urinary β2 microglobulin and urinary *N*-acetyl-beta-glucosaminidase levels increased [594 mg/dl (normal, <5 mg/dl), 886.4 µg/dl (normal, 5-200 µg/dl) and 14.8 U/l (normal, <5 U/l), respectively]. The patient was diagnosed with interstitial nephritis. In patients with chronic kidney diseases, the eGFR in those with grade 2 or 3 renal dysfunction is 30-59 ml/min/1.73 m^2^, or 15-30 ml/min/1.73 m^2^, respectively. The patient began to take 20 mg prednisolone (generic drug; oral route) daily, and his renal function gradually improved.

At 4 months after the combination treatment was suspended, no recurrences were observed (Fig. 1G-I). The patient's tumor marker levels remained within normal limits: AFP, 1.1 ng/ml; AFP-L3, <0.5%; and PIVKA-II, 11 mAU/ml. His serum creatinine, albumin levels and eGFR were 1.60 mg/dl, 2.9 g/dl and 32.9 ml/min/1.73 m^2^, respectively. His urinary protein level was 90 mg/dl. His leg edema disappeared although he still took 5 mg prednisolone daily. The authors plan to follow-up the patient carefully.

## Discussion

The present study reports the case of a male patient in his 80s who consumed alcohol, with unresectable advanced-stage HCC who received atezolizumab plus bevacizumab for 6 months and achieved a CR. His performance status and Child-Pugh were ECOG PS-1 and grade A, respectively. The case described herein suggests that it is critical for patients with HCC and Child-Pugh A to continue therapy for HCC, even if they are older.

It has been demonstrated that in patients with unresectable HCC (median age, 64 years; range, 56-71 years), atezolizumab combined with bevacizumab results in improved overall and progression-free survival outcomes than sorafenib (4). Hosoda *et al* (16) reported successful multidisciplinary treatment with a CR to atezolizumab plus bevacizumab in a 90-year-old patient with HCC recurrence. Hatanaka *et al* (10) also reported that treatment with atezolizumab plus bevacizumab had an efficacy comparable to that of treatment with lenvatinib in HCC patients aged ≥80 years, and a CR was observed in 7.6% (7/92) of the patients.

ICI monotherapy or the immune-based combinations are associated with an improved survival, irrespective of the ECOG PS-0 or PS-1 status (17). HCC is one of the male-dominant cancers (18-20). Santoni *et al* (21) reported the sex difference in the efficacy of ICIs among cancer patients. However, further studies on this point are required among patients with HCC.

For the majority of patients with advanced-stage HCC, systemic therapy or BSC can be selected (1-3). A recent meta-analysis demonstrated that ICI therapy in the Child-Pugh B grade group was safe and was associated with a significant number of radiological responses, although survival outcomes were superior in the Child-Pugh A grade group (22). The development of more effective therapies is required for patients with advanced-stage HCC with a worse liver function than those with Child-Pugh A grade (23,24).

In case described in the present study, nephrotoxicity was observed during combination therapy with atezolizumab plus bevacizumab, although a renal biopsy was not performed. Nephrotoxicity, which can cause proteinuria, is one of the complications of both chemotherapy and immunotherapy (25-27).

Combination therapy comprising atezolizumab plus bevacizumab has been reported to be safe and widely effective (4,28,29). This combination may also play critical roles as systemic adjuvant treatment in HCC (30,31). Trans-arterial chemoembolization plus ICI may play a role in the treatment of patients with HCC (32).

In conclusion, as demonstrated in the present study, combination therapy comprising atezolizumab plus bevacizumab is effective for elderly patients with unresectable HCC. Careful attention should be paid to adverse events, including immune-related adverse events during and following combination treatment in elderly patients.

## Figures and Tables

**Table I:** Laboratory data at the first visit of the patient to the hospital.

Items	Values	Items	Values
White blood cell counts	5,400/µl	Blood urea nitrogen	4.2 mg/dl
Hemoglobin	12.0 g/dl	Creatinine	1.12 mg/dl
Platelet counts	235,000/µl	eGFR	48.7 ml/min/1.73 m^2^
PT	93%	Glucose	102 mg/dl
INR	1.04	Hemoglobin A1c	6.4%
AST	85 IU/l	HBsAg	Negative
ALT	18 IU/l	anti-HBs	Positive
ALP	103 IU/l	anti-HBc	Positive
γ-GTP	690 IU/l	anti-HCV	Negative
Total bilirubin	0.42 mg/dl	ANA	<40
Total protein	6.8 g/dl	AFP	22,771 ng/ml
Albumin	3.7 g/dl	AFP-L3	66.0%
Total cholesterol	143 mg/dl	PIVKA-II	27 mAU/ml

PT, prothrombin time; INR, international normalized ratio; AST, aspartate aminotransferase; ALT, alanine aminotransferase; ALP, alkaline phosphatase; γ-GTP, γ-glutamyl transpeptidase; eGFR, estimated glomerular filtration rate; HBsAg, hepatitis B surface antigen; anti-HBc, anti-hepatitis B core antibody; anti-HCV, anti-hepatitis C virus antibody; ANA, anti-nuclear antibody; AFP, α-fetoprotein; AFP-L3, lectin-reactive profile of AFP; PIVKA-II, protein induced by vitamin K absence or antagonist II.

## Data Availability

The datasets used and/or analyzed during the current study are available from the corresponding author upon reasonable request.

## References

[1] Omata M, Cheng AL, Kokudo N, Kudo M, Lee JM, Jia J, Tateishi R, Han KH, Chawla YK, Shiina S (2017). Asia-Pacific clinical practice guidelines on the management of hepatocellular carcinoma: A 2017 update. Hepatol Int.

[2] (2018). EASL Clinical Practice Guidelines: Management of hepatocellular carcinoma. J Hepatol.

[3] Marrero JA, Kulik LM, Sirlin CB, Zhu AX, Finn RS, Abecassis MM, Roberts LR, Heimbach JK (2018). Diagnosis, staging, and management of hepatocellular carcinoma: 2018 practice guidance by the American association for the study of liver diseases. Hepatology.

[4] Finn RS, Qin S, Ikeda M, Galle PR, Ducreux M, Kim TY, Kudo M, Breder V, Merle P, Kaseb AO (2020). Atezolizumab plus bevacizumab in unresectable hepatocellular carcinoma. N Engl J Med.

[5] Kelley RK, Sangro B, Harris W, Ikeda M, Okusaka T, Kang YK, Qin S, Tai DW, Lim HY, Yau T (2021). Safety, efficacy, and pharmacodynamics of tremelimumab plus durvalumab for patients with unresectable hepatocellular carcinoma: Randomized expansion of a phase I/II study. J Clin Oncol.

[6] Okuda K, Obata H, Nakajima Y, Ohtsuki T, Okazaki N, Ohnishi K (1984). Prognosis of primary hepatocellular carcinoma. Hepatology.

[7] Hanazaki K, Kajikawa S, Shimozawa N, Shimada K, Hiraguri M, Koide N, Adachi W, Amano J (2001). Hepatic resection for hepatocellular carcinoma in the elderly. J Am Coll Surg.

[8] Sato M, Tateishi R, Yasunaga H, Horiguchi H, Yoshida H, Matsuda S, Koike K (2012). Mortality and morbidity of hepatectomy, radiofrequency ablation, and embolization for hepatocellular carcinoma: A national survey of 54,145 patients. J Gastroenterol.

[9] Marta GN, da Fonseca LG, Braghiroli MI, Moura F, Hoff PM, Sabbaga J (2021). Efficacy and safety of sorafenib in elderly patients with advanced hepatocellular carcinoma. Clinics (Sao Paulo).

[10] Hatanaka T, Kakizaki S, Hiraoka A, Tada T, Hirooka M, Kariyama K, Tani J, Atsukawa M, Takaguchi K, Itobayashi E

[11] Ishikawa T, Yamazaki S, Sato R, Jimbo R, Kobayashi Y, Sato T, Iwanaga A, Sano T, Yokoyama J, Honma T (2024). Efficacy of adding locoregional therapy in non-complete remission hepatocellular carcinoma treated with atezolizumab plus bevacizumab: A preliminary study. Anticancer Res.

[12] Gatson NTN, Makary M, Bross SP, Vadakara J, Maiers T, Mongelluzzo GJ, Leese EN, Brimley C, Fonkem E, Mahadevan A (2020). Case series review of neuroradiologic changes associated with immune checkpoint inhibitor therapy. Neurooncol Pract.

[13] Eisenhauer EA, Therasse P, Bogaerts J, Schwartz LH, Sargent D, Ford R, Dancey J, Arbuck S, Gwyther S, Mooney M (2009). New response evaluation criteria in solid tumours: Revised RECIST guideline (version 1.1). Eur J Cancer.

[14] Lencioni R, Llovet JM (2010). Modified RECIST (mRECIST) assessment for hepatocellular carcinoma. Semin Liver Dis.

[15] Minagawa M, Ikai I, Matsuyama Y, Yamaoka Y, Makuuchi M (2007). Staging of hepatocellular carcinoma: Assessment of the Japanese TNM and AJCC/UICC TNM systems in a cohort of 13,772 patients in Japan. Ann Surg.

[16] Hosoda K, Toshima T, Takahashi J, Yonemura Y, Hisamatsu Y, Hirose K, Masuda T, Motomura Y, Abe T, Ando Y (2023). Successful multidisciplinary treatment with complete response to atezolizumab plus bevacizumab in a 90-year-old patient with hepatocellular carcinoma recurrence. Int Cancer Conf J.

[17] Mollica V, Rizzo A, Marchetti A, Tateo V, Tassinari E, Rosellini M, Massafra R, Santoni M, Massari F (2023). The impact of ECOG performance status on efficacy of immunotherapy and immune-based combinations in cancer patients: The MOUSEION-06 study. Clin Exp Med.

[18] Shiratori Y, Shiina S, Zhang PY, Ohno E, Okudaira T, Payawal DA, Ono-Nita SK, Imamura M, Kato N, Omata M (1997). Does dual infection by hepatitis B and C viruses play an important role in the pathogenesis of hepatocellular carcinoma in Japan?. Cancer.

[19] Chiu CM, Yeh SH, Chen PJ, Kuo TJ, Chang CJ, Chen PJ, Yang WJ, Chen DS (2007). Hepatitis B virus X protein enhances androgen receptor-responsive gene expression depending on androgen level. Proc Natl Acad Sci USA.

[20] Kanda T, Steele R, Ray R, Ray RB (2008). Hepatitis C virus core protein augments androgen receptor-mediated signaling. J Virol.

[21] Santoni M, Rizzo A, Mollica V, Matrana MR, Rosellini M, Faloppi L, Marchetti A, Battelli N, Massari F (2022). The impact of gender on The efficacy of immune checkpoint inhibitors in cancer patients: The MOUSEION-01 study. Crit Rev Oncol Hematol.

[22] Xie E, Yeo YH, Scheiner B, Zhang Y, Hiraoka A, Tantai X, Fessas P, de Castro T, D'Alessio A, Fulgenzi CAM (2023). Immune checkpoint inhibitors for child-pugh class B advanced hepatocellular carcinoma: A systematic review and meta-analysis. JAMA Oncol.

[23] Tanaka T, Hiraoka A, Tada T, Hirooka M, Kariyama K, Tani J, Atsukawa M, Takaguchi K, Itobayashi E, Fukunishi S (2022). Therapeutic efficacy of atezolizumab plus bevacizumab treatment for unresectable hepatocellular carcinoma in patients with Child-Pugh class A or B liver function in real-world clinical practice. Hepatol Res.

[24] Ramaswamy A, Kulkarni A, John G, Rauthan A, Patil S, Duseja A, Talwar V, Rajappa SJ, Ghadyalpatil N, Wadhawan M (2023). Survival of trial-like and non-trial-like patients with immunotherapy in advanced hepatocellular carcinoma in real world: A collaborative multicenter indian study (IMHEP). JCO Glob Oncol.

[25] Barakat RK, Singh N, Lal R, Verani RR, Finkel KW, Foringer JR (2007). Interstitial nephritis secondary to bevacizumab treatment in metastatic leiomyosarcoma. Ann Pharmacother.

[26] Xipell M, Victoria I, Hoffmann V, Villarreal J, García-Herrera A, Reig O, Rodas L, Blasco M, Poch E, Mellado B, Quintana LF (2018). Acute tubulointerstitial nephritis associated with atezolizumab, an anti-programmed death-ligand 1 (pd-l1) antibody therapy. Oncoimmunology.

[27] Jagieła J, Bartnicki P, Rysz J (2021). Nephrotoxicity as a complication of chemotherapy and immunotherapy in the treatment of colorectal cancer, melanoma and non-small cell lung cancer. Int J Mol Sci.

[28] Gao X, Zhao R, Ma H, Zuo S (2023). Efficacy and safety of atezolizumab plus bevacizumab treatment for advanced hepatocellular carcinoma in the real world: A single-arm meta-analysis. BMC Cancer.

[29] Kulkarni AV, Tevethia H, Kumar K, Premkumar M, Muttaiah MD, Hiraoka A, Hatanaka T, Tada T, Kumada T, Kakizaki S (2023). Effectiveness and safety of atezolizumab-bevacizumab in patients with unresectable hepatocellular carcinoma: A systematic review and meta-analysis. EClinicalMedicine.

[30] Rizzo A, Ricci AD, Brandi G (2020). Systemic adjuvant treatment in hepatocellular carcinoma: Tempted to do something rather than nothing. Future Oncol.

[31] Qin S, Chen M, Cheng AL, Kaseb AO, Kudo M, Lee HC, Yopp AC, Zhou J, Wang L, Wen X (2023). Atezolizumab plus bevacizumab versus active surveillance in patients with resected or ablated high-risk hepatocellular carcinoma (IMbrave050): A randomised, open-label, multicentre, phase 3 trial. Lancet.

[32] Rizzo A, Ricci AD, Brandi G (2022). Trans-Arterial chemoembolization plus systemic treatments for hepatocellular carcinoma: An update. J Pers Med.

